# Cellular and Humoral Immune Responses in Anti-N Seropositive and Seronegative Vaccinated Participants: Insights from the Bialystok PLUS Study

**DOI:** 10.3390/vaccines14090813

**Published:** 2026-09-16

**Authors:** Gabriela Trojan, Anna Moniuszko-Malinowska, Justyna Adamczuk, Anna Citko-Rojewska, Maciej Alimowski, Piotr Czupryna, Kiara Sequeira, Hami Nguyen, Justyna Dunaj-Małyszko, Paweł Sowa, Marlena Dubatówka, Magda Łapińska, Sebastian Sołomacha, Małgorzata Kazberuk, Magdalena Chlabicz, Łukasz Kiszkiel, Łukasz Szczerbiński, Piotr Paweł Laskowski, Karol Adam Kamiński

**Affiliations:** 1 Department of Infectious Diseases and Neuroinfection, Medical University of Bialystok, Zurawia 14, 15-540 Bialystok, Poland; 2 Clinical Research Centre, Medical University of Bialystok, M. Sklodowskiej-Curie 24a, 15-276 Bialystok, Poland; 3 Doctoral School of Social Sciences, University of Bialystok, 15-403 Bialystok, Poland; 4 The Faculty of Medicine with the Division of Dentistry and the Division of Medical Education in English, Medical University of Bialystok, Jana Kilińskiego 1, 15-089 Białystok, Poland; 5 Department of Population Medicine and Lifestyle Diseases Prevention, Medical University of Bialystok, 15-269 Bialystok, Poland; 6 Society and Cognition Unit, University of Bialystok, 15-403 Bialystok, Poland; 7 Department of Endocrinology, Diabetology and Internal Medicine, Medical University of Bialystok, M. Sklodowskiej-Curie 24A, 15-276 Bialystok, Poland

**Keywords:** SARS-CoV-2, hybrid immunity, cellular immunity, humoral response, vaccination, antibodies, COVID-19

## Abstract

**Background:** In the post-pandemic era, SARS-CoV-2 immunity is increasingly shaped by vaccination and previous infection. Understanding the relationship between cellular and humoral immune responses is important for characterizing immunity in vaccinated populations. **Methods:** This study included 629 participants from the Bialystok PLUS cohort, stratified according to anti-N serostatus into anti-N-seronegative (n = 159) and anti-N-seropositive (n = 470) groups. Cellular immune response measurements were available for a subset of 246 participants. Humoral response was assessed using anti-S and anti-N antibodies, while cellular response (OC) was evaluated by interferon-gamma (IFN-γ) release following SARS-CoV-2 antigen stimulation. Group differences were assessed using the Mann–Whitney U test, and associations were evaluated using Spearman’s rank correlations with Benjamini–Hochberg correction. Multivariable linear regression was used to assess the association between anti-N serostatus and cellular response after adjustment for age and sex. **Results:** Anti-N-seropositive participants had significantly higher cellular responses than anti-N-seronegative participants (median 1752.3 vs. 771.1; *p* < 0.001). After adjustment for age and sex, anti-N seropositivity remained significantly associated with higher cellular response, corresponding to a 3.57-fold higher geometric mean of OC + 1 (95% CI: 1.90–6.73; *p* < 0.001). Anti-S antibody levels showed moderate positive correlations with cellular response in both anti-N-seronegative and anti-N-seropositive participants (ρ = 0.45 and ρ = 0.37, respectively), and the association remained significant after adjustment for age, sex, and anti-N serostatus. No significant associations were observed between cellular response and anti-N antibody levels or routine laboratory parameters after correction for multiple comparisons. **Conclusions:** Anti-N seropositivity was associated with higher SARS-CoV-2-specific cellular immune response among vaccinated participants, and this association remained significant after adjustment for age and sex. The moderate association between anti-S antibody levels and cellular response suggests that humoral and cellular measures provide related but complementary information on SARS-CoV-2-specific immunity. Routine laboratory parameters were not significantly associated with cellular immune response in this cohort.

## 1. Introduction

Five years after its emergence, SARS-CoV-2 remains an evolving public health challenge despite the transition from the acute pandemic phase to endemic circulation. Recently, World Health Organization (WHO) reported that, despite stabilization of mortality rates, global health gains over the past two decades are slowing due to long-term disruptions and COVID-19-related sequelae [1]. We are no longer fighting a novel pathogen in a naïve population; instead, we are navigating a complex landscape where the virus continues to circulate widely among billions of people with varying degrees of immune memory [2]. This shift has altered clinical priorities, with increasing emphasis on understanding the quality, durability, and breadth of immune responses in populations where hybrid immunity has become increasingly common [3].

Recent epidemiological data from Poland reported by Adamczuk et al. (2025) demonstrated how pervasive the virus has become. Their study demonstrated that seroprevalence of anti-spike antibodies in the general population has reached approximately 92.5%, a testament to both the reach of vaccination campaigns and widespread transmission [4]. Perhaps more illustrative is that almost 70% of the population has antibodies against the nucleocapsid, the antigen specific to natural infection [4]. Crucially, a substantial proportion of these individuals had no documented history of COVID-19. These findings suggest that a considerable proportion of SARS-CoV-2 infections were not clinically documented [4,5].

However, the presence of antibodies tells only half the story. While high titers of neutralizing antibodies represent the main barrier to initial infection, these are notoriously transient. Studies have consistently demonstrated that antibody levels decline rapidly during the months after vaccination or infection, a decline further hastened by the emergence of immune-evasive Omicron sublineages [6,7]. If serology were the sole guide, the population would appear increasingly vulnerable over time. Yet protection against severe disease, hospitalization, and death remains robust [8]. This decoupling of infection risk from disease severity points to the critical role of the adaptive immune system’s second arm: the cellular response, mediated by T lymphocytes [9].

By contrast to antibodies, which intercept free virus, T cells are responsible for identifying and eliminating infected cells, thereby abrogating viral replication and preventing systemic dissemination. Overall, T-cell immunity against coronaviruses is generally more durable than humoral immunity and less sensitive to viral mutations since T cells recognize a greater array of viral epitopes that are preserved across variants [10,11]. Assessment of SARS-CoV-2 antigen-stimulated IFN-γ release may provide complementary information on the cellular immune response [12].

Despite its clinical importance, cellular immunity is rarely quantified in routine practice. Methods used for detailed assessment of T-cell function and phenotype are complex, relatively costly, and difficult to standardize for high-throughput clinical use. Consequently, assessment of cellular immunity is not routinely performed in clinical practice [13].

Furthermore, it remains unclear whether routine hematological parameters, which change dynamically during acute infection, could serve as stable biomarkers for the magnitude of this cellular memory in healthy, vaccinated cohorts. Previous studies conducted during acute SARS-CoV-2 infection have demonstrated associations between disease severity, inflammatory cytokine profiles, and distinct cellular immune phenotypes [14,15]. However, whether such relationships persist beyond the acute phase, particularly in vaccinated individuals with previous SARS-CoV-2 exposure, remains less well established.

Whereas the current literature extensively documented the prognostic value of markers such as the neutrophil-to-lymphocyte ratio or platelet indices in the acute phase of COVID-19, strongly correlating with cytokine storm intensity and mortality, their utility in the post-convalescent or stable post-vaccination period remains less well established [16]. Whether routine laboratory parameters are associated with SARS-CoV-2-specific cellular responses in vaccinated individuals remains unclear.

Therefore, this study aimed to characterize SARS-CoV-2-specific cellular immune responses in vaccinated participants according to anti-N serostatus, to examine the relationship between antigen-stimulated IFN-γ responses and quantitative humoral markers, particularly anti-S antibodies, and to assess whether routine laboratory parameters are associated with cellular immune responsiveness.

## 2. Materials and Methods

### 2.1. Study Population and Group Definitions

The study included vaccinated participants from the Bialystok PLUS Study, a population-based cohort conducted in northeastern Poland. Participants were categorized according to their anti-nucleocapsid (anti-N) serological status.

A total of 629 vaccinated participants underwent serological assessment and were stratified according to anti-N serological status into anti-N seronegative (n = 159) and anti-N seropositive (n = 470) groups. Cellular immune response measurements (SARS-CoV-2 antigen-stimulated IFN-γ release) were available for a subset of participants (n = 246); therefore, analyses involving cellular immunity were performed only in this subgroup.

Vaccination-related information available in the study database included vaccination status, and vaccine manufacturer. Detailed information on the timing of vaccination or previous SARS-CoV-2 exposure relative to blood sampling was not sufficiently available for analysis. The criteria determining which participants underwent cellular immune response testing were not recorded in the available database.

### 2.2. Laboratory Assessments

Humoral immune response was evaluated by measuring SARS-CoV-2-specific antibodies. Anti-N antibodies were detected using the Elecsys Anti-SARS-CoV-2 assay (Roche Diagnostics, Rotkreuz, Switzerland) on the fully automated cobas platform (Roche Diagnostics International Ltd., Rotkreuz, Switzerland). Results were reported as reactive or non-reactive, together with a cutoff index (COI; sample signal/cutoff), with a COI ≥ 1.0 indicating a reactive result. Anti-S IgG antibodies were measured using the LIAISON SARS-CoV-2 S1/S2 assay (DiaSorin, Saluggia, Italy) on the fully automated LIAISON XL platform (DiaSorin S.p.A., Saluggia, Italy). Results were expressed in AU/mL, with values >15.0 AU/mL considered positive according to the manufacturer’s instructions. The assay has been reported to show 94.4% positive agreement with the plaque reduction neutralization test (PRNT90).

Cellular immune response (OC) was assessed by measuring SARS-CoV-2 antigen-stimulated interferon-gamma (IFN-γ) release using the Quan-T-Cell ELISA (EUROIMMUN; catalog no. EQ 6841-9601). Whole blood samples were stimulated with Quan-T-Cell SARS-CoV-2 reagents (EUROIMMUN; catalog no. ET 2606-3003), and IFN-γ concentrations were measured using the EUROIMMUN Analyzer I-2P (EUROIMMUN Medizinische Labordiagnostika AG, Lübeck, Germany) according to the manufacturer’s instructions. Hereafter, the SARS-CoV-2 antigen-stimulated IFN-γ response is referred to as the cellular response (OC). Cellular immune response measurements were available only for participants for whom the corresponding whole-blood samples were collected and analyzed using the cellular assay.

All laboratory assays were performed according to the manufacturers’ instructions.

Routine laboratory parameters included CRP, WBC, lymphocyte count/percentage, PLT, CK, LDH, ALT, AST, bilirubin, GGT, glucose, total protein, albumin, ferritin, creatinine, sodium, potassium, IL-6, D-dimer, fibrinogen, anti-HCV, HBsAg, total cholesterol, HDL cholesterol, LDL cholesterol, triglycerides, and vitamin D.

### 2.3. Data Processing

Data were checked for consistency and converted to appropriate formats for statistical analysis. Missing or invalid values were treated as missing and were not imputed. Correlation analyses were performed using pairwise complete observations.

#### 2.3.1. Statistical Analysis

All statistical analyses were performed using R software (version 4.5.1; R Core Team, Vienna, Austria), with the use of the tidyverse and dplyr packages for data processing and ggstatsplot for statistical visualization.

Comparisons between groups were conducted using the Mann–Whitney U test. Effect sizes were reported as rank-biserial correlation coefficients, along with their corresponding confidence intervals.

As IFN-γ concentrations were not normally distributed, non-parametric statistical methods were applied throughout the analyses.

Associations between cellular response (OC) and humoral markers (anti-S and anti-N) were evaluated using Spearman’s rank correlation coefficient with two-sided testing.

For analyses involving multiple laboratory parameters, the Benjamini–Hochberg false discovery rate (FDR) correction was applied to adjust for multiple comparisons.

Pairwise deletion was used in correlation analyses, including only observations with complete data for each pair of variables. A minimum sample size of ≥10 observations was required for correlation analyses involving laboratory parameters. A two-sided *p*-value < 0.05 was considered statistically significant.

#### 2.3.2. Ethical Issues

Ethical approval for the study was obtained from the Ethics Committee of the Medical University of Bialystok, Poland (approval no. APK.002.346.2020). All procedures were conducted in accordance with the principles of the Declaration of Helsinki and its subsequent amendments. Participation was voluntary, and written informed consent was obtained from all participants. Prior to enrollment, all study procedures were thoroughly explained to each participant.

## 3. Results

### 3.1. Study Population and Baseline Characteristics

The study included 629 participants, of whom 159 were anti-N seronegative and 470 were anti-N seropositive. The mean age was 51.9 years, and 52.8% of participants were female. Cellular immune response measurements were available for 246 participants (39.1%), including 46 anti-N-seronegative and 200 anti-N-seropositive participants. Detailed demographic, vaccination-related, serological, and cellular characteristics are presented in Table 1.

To assess potential differences between participants with and without available cellular response measurements, these two groups were compared. Age and sex distributions were similar between the groups, whereas anti-N seropositivity was more frequent among participants with available OC measurements (81.3% vs. 70.5%; BH-adjusted *p* = 0.006).

**Figure 1:**
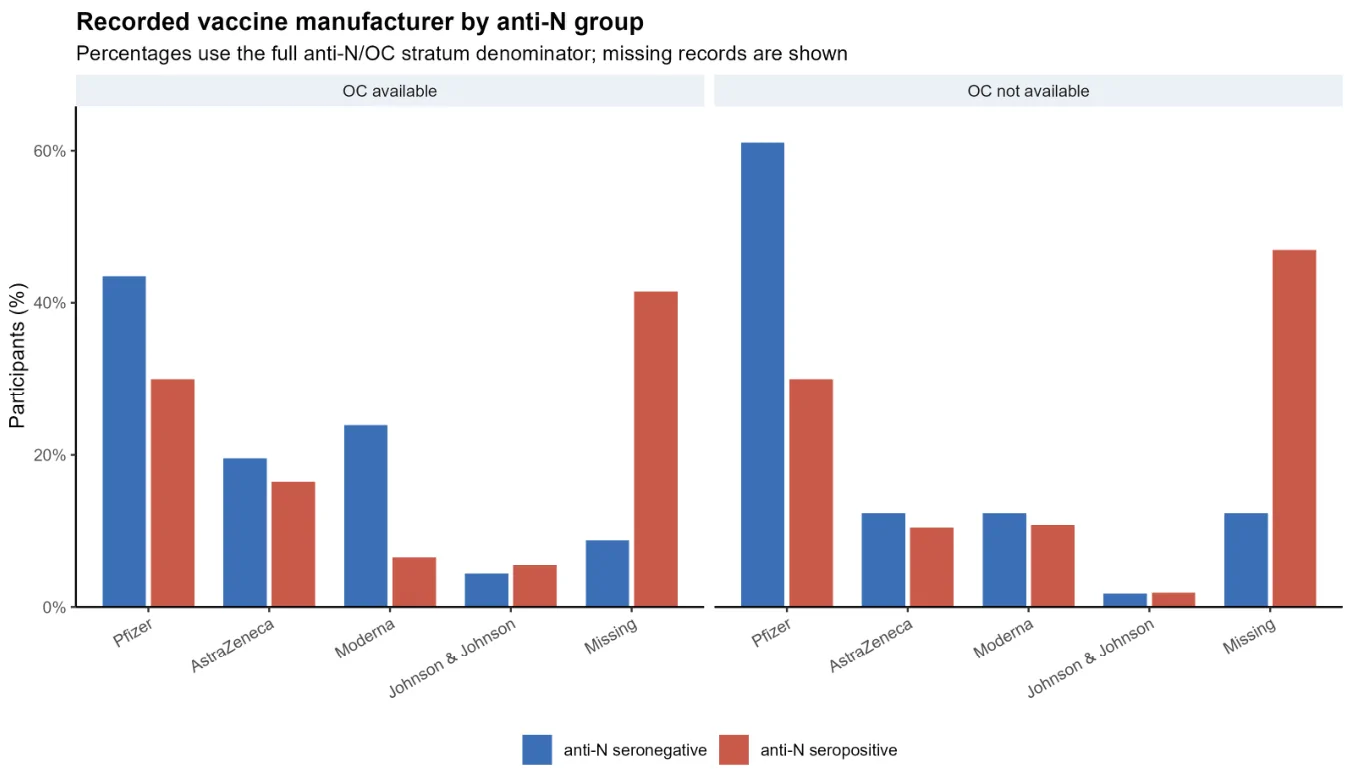
Distribution of recorded COVID-19 vaccine manufacturers according to availability of cellular immune response measurements.

Among the 629 participants, information on vaccine manufacturer was available for 401 (63.8%). Pfizer was the most frequently recorded vaccine manufacturer (230/629, 36.6%), followed by AstraZeneca (84/629, 13.4%), Moderna (67/629, 10.7%), and Johnson & Johnson (20/629, 3.2%). Information on vaccine manufacturer was unavailable for 228 participants (36.2%). The distribution of recorded vaccine manufacturers according to the availability of cellular immune response measurements is shown in Figure 1.

### 3.2. Cellular Immune Response

**Figure 2:**
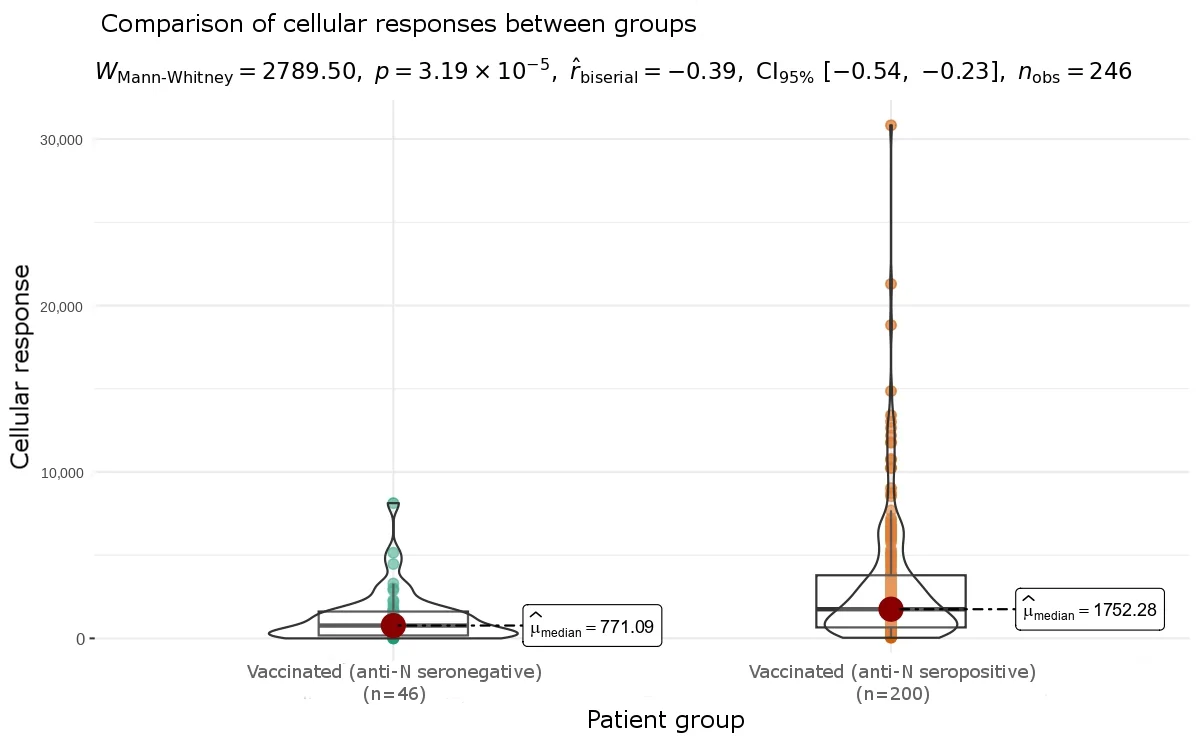
Comparison of cellular immune response between groups. Cellular immune response (OC) in vaccinated anti-N-seronegative and anti-N-seropositive participants. Data are presented as violin plots with embedded boxplots, illustrating the distribution, median, and interquartile range. Individual data points are shown. Teal and orange indicate anti-N-seronegative and anti-N-seropositive participants, respectively. Red dots indicate group medians, and dashed lines connect the median markers with their corresponding numerical values.

The cellular immune response (OC) differed significantly between groups. Anti-N seropositive participants exhibited markedly higher OC values compared to anti-N seronegative (median 1752.3 vs. 771.1). This difference was statistically significant (Mann–Whitney U test: W = 2789.5, *p* = 3.19 × 10^−5^), with a moderate effect size (rank-biserial correlation r = −0.39, 95% CI: −0.54 to −0.23) (Figure 2).

Descriptive statistics further supported this finding, with higher mean OC values in the anti-N-seropositive group (3124.3 ± 3999.4) than in the anti-N-seronegative group (1245.6 ± 1570.2).

A significantly higher cellular response was observed in the anti-N-seropositive group compared to the anti-N-seronegative group (Mann–Whitney U test: W = 2789.5, *p* = 3.19 × 10^−5^; rank-biserial correlation r = −0.39, 95% CI: −0.54 to −0.23). Median values were 771.1 in the anti-N-seronegative group and 1752.3 in the anti-N-seropositive group.

### 3.3. Humoral Immune Response

As expected from the group definition, anti-N antibody status was negative in all anti-N-seronegative participants and positive in all anti-N-seropositive participants. Quantitative anti-N levels were substantially higher in the anti-N-seropositive group (mean 94.22 ± 76.89; median 73.38) than in the anti-N-seronegative group (mean 0.141 ± 0.184; median 0.079).

Anti-S antibodies were detected in all participants (100% positivity in both groups). Anti-S antibody levels were higher in the anti-N-seropositive group than in the anti-N-seronegative group (median 1460 vs. 1240), although substantial inter-individual variability was observed in both groups (mean 4985.2 ± 9367.5 vs. 3720.0 ± 9176.5, respectively).

### 3.4. Associations Between Cellular and Humoral Responses

**Figure 3:**
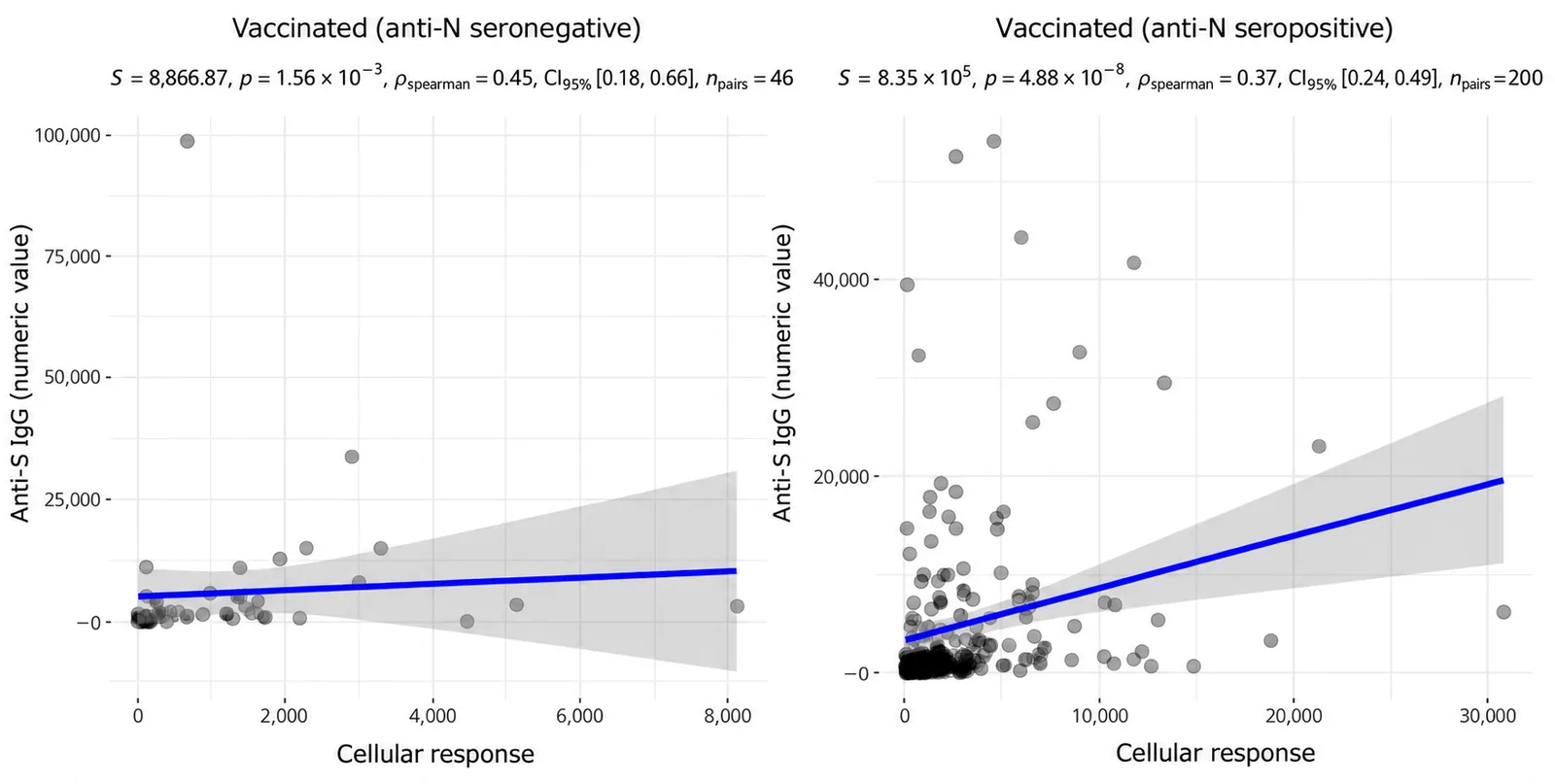
Association between cellular and humoral immune responses (Scatter plots illustrating the relationship between cellular immune response (OC) and anti-S antibody levels in vaccinated individuals anti-N-seronegative (**left panel**) and anti-N-seropositive (**right panel**). Each point represents an individual participant. The blue line indicates the fitted regression line, with the shaded area representing the 95% confidence interval).

A significant positive correlation was observed between cellular response (OC) and anti-S antibody levels in both groups. In anti-N-seronegative participants, the correlation was moderate (Spearman’s ρ = 0.45, *p* = 1.56 × 10^−3^, FDR-adjusted *p* = 0.0031; 95% CI: 0.18–0.66; n = 46). A similar association was observed in anti-N-seropositive participants (ρ = 0.37, *p* = 4.88 × 10^−8^, FDR-adjusted *p* = 9.76 × 10^−8^; 95% CI: 0.24–0.49; n = 200) (Figure 3).

**Figure 4:**
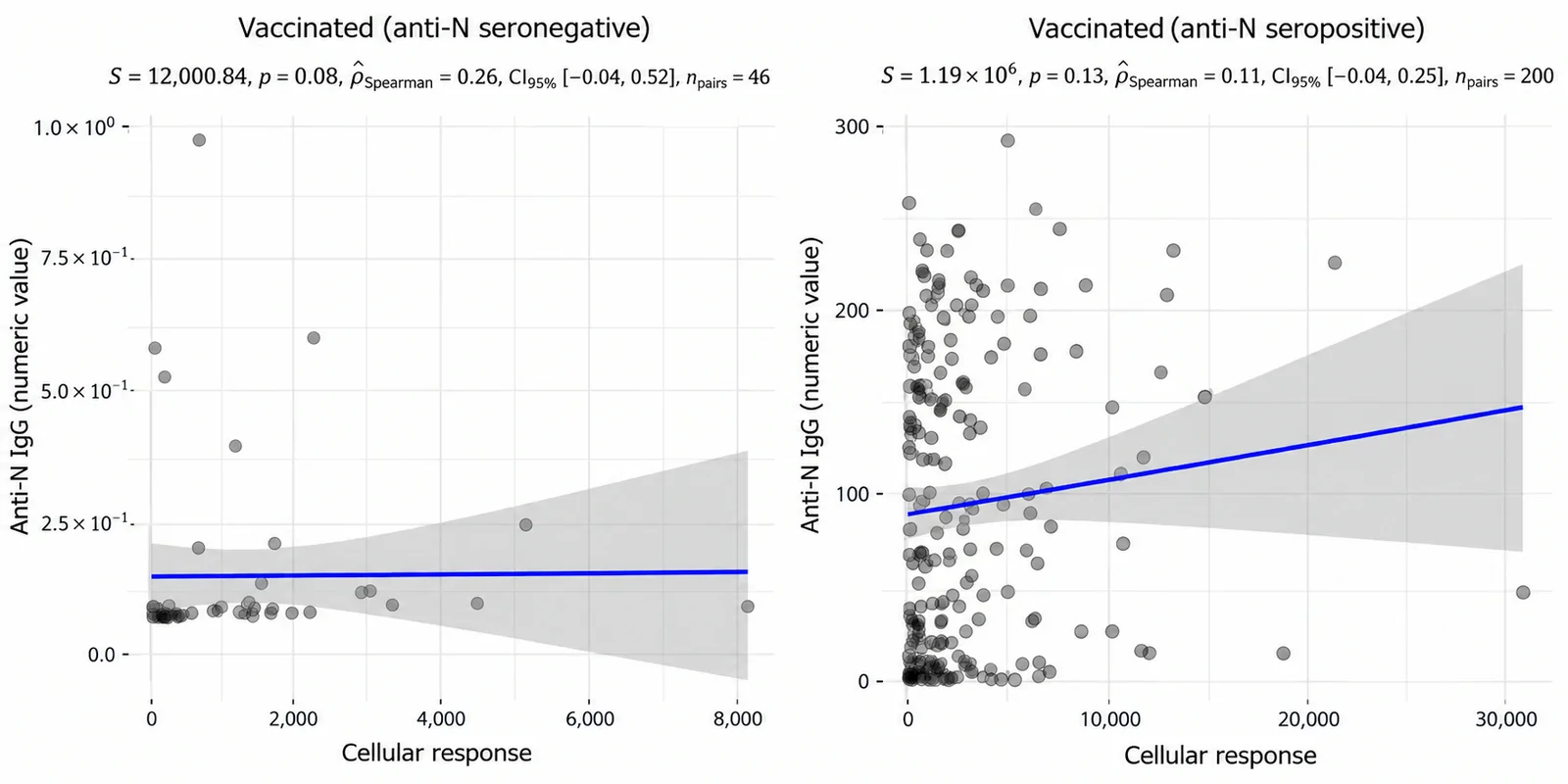
Lack of association between cellular response and anti-N antibody levels (Scatter plots illustrating the relationship between cellular immune response (OC) and anti-N antibody levels in vaccinated individuals anti-N seronegative (**left panel**) and anti-N seropositive (**right panel**). Each point represents an individual participant. The blue line indicates the fitted regression line, with the shaded area representing the 95% confidence interval).

In contrast, no statistically significant correlations were observed between OC and anti-N antibody levels in either group. In anti-N-seronegative participants, the correlation was weak and did not reach statistical significance (Spearman’s ρ = 0.26, *p* = 0.081; 95% CI: −0.04–0.52; n = 46). Similarly, no significant association was observed in anti-N-seropositive participants (ρ = 0.11, *p* = 0.128; 95% CI: −0.04–0.25; n = 200) (Figure 4).

To further evaluate the association between anti-N serostatus and cellular immune response, multivariable linear regression analysis was performed using log-transformed cellular response values [log(OC + 1)] as the dependent variable. After adjustment for age and sex, anti-N-seropositive participants had significantly higher cellular responses than anti-N-seronegative participants, corresponding to a 3.57-fold higher geometric mean of OC + 1 (95% CI: 1.90–6.73; *p* < 0.001; n = 246).

The association remained significant after additional adjustment for vaccine manufacturer, with a 4.25-fold higher geometric mean of OC + 1 in anti-N-seropositive participants (95% CI: 2.10–8.61; n = 159). This model included fewer participants (n = 159) because of missing vaccine manufacturer data and should therefore be interpreted with caution.

We also assessed the association between anti-S antibody levels and cellular response. After adjustment for age, sex, and anti-N serostatus, each two-fold increase in anti-S antibody concentration was associated with a 1.29-fold higher cellular response (geometric mean ratio for OC + 1 = 1.29; 95% CI: 1.18–1.41; *p* < 0.001; n = 246). There was no evidence that the strength of this association differed between anti-N-seronegative and anti-N-seropositive participants (*p* for interaction = 0.360). The association remained consistent in models additionally adjusted for vaccine manufacturer, although these analyses included fewer participants because of missing data.

### 3.5. Associations with Laboratory Parameters

No statistically significant correlations between OC and routine laboratory parameters were identified after correction for multiple comparisons (Benjamini–Hochberg FDR < 0.05) in either group.

In the anti-N-seropositive group, several weak associations were observed prior to correction, including correlations with lymphocyte percentage (ρ ≈ 0.21) and granulocyte percentage (ρ ≈ −0.20), as well as selected platelet indices (e.g., MPV, PLCR), with effect sizes ranging from small to moderate (|ρ| ≈ 0.13–0.29). However, these associations did not remain significant after FDR adjustment.

**Figure 5:**
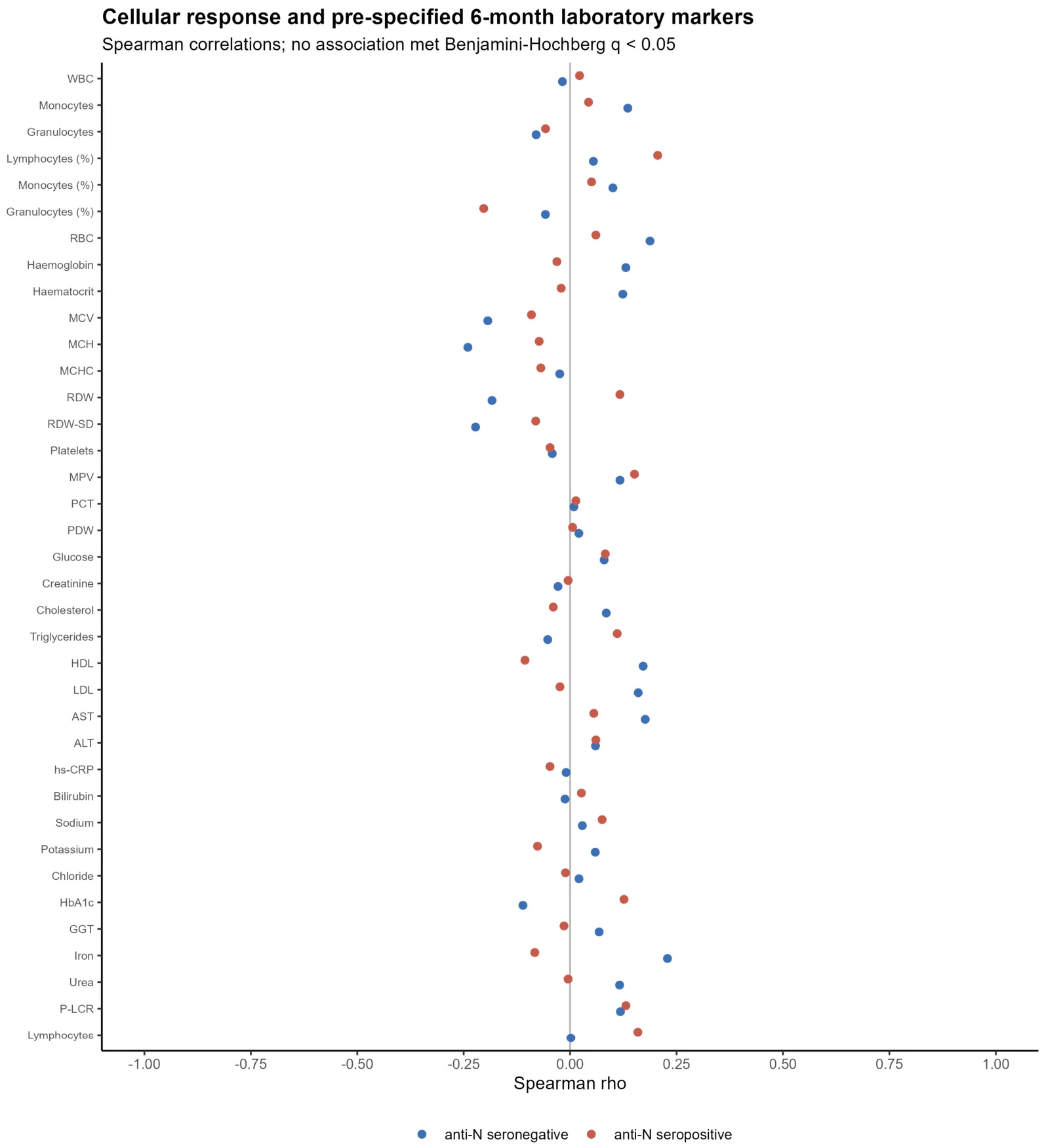
Associations between SARS-CoV-2 antigen-stimulated IFN-γ response (OC) and routine laboratory parameters. Spearman correlation coefficients are shown for the analyzed laboratory parameters in the anti-N-seronegative and anti-N-seropositive groups. None of the associations remained statistically significant after Benjamini–Hochberg correction for multiple comparisons.

In the anti-N-seronegative group, no meaningful associations were detected, likely due to smaller sample sizes in pairwise analyses (n = 46). The complete set of Spearman correlation coefficients for the analyzed laboratory parameters is shown in Figure 5.

## 4. Discussion

Anti-N seropositivity is commonly used as a serological marker of previous SARS-CoV-2 exposure; however, the absence of detectable anti-N antibodies does not definitively exclude previous infection because anti-N responses may wane over time, particularly after asymptomatic or mild infection. Previous studies have also described SARS-CoV-2-exposed individuals who remained seronegative despite detectable differences in SARS-CoV-2-specific T-cell responses, further supporting the need for caution when interpreting anti-N seronegativity as definitive evidence of absence of prior infection [17]. Therefore, in the present study, anti-N serostatus was used as an operational marker of previous SARS-CoV-2 exposure rather than as definitive evidence of infection history. Within this framework, we examined SARS-CoV-2 antigen-stimulated IFN-γ responses in vaccinated participants according to anti-N serostatus and further evaluated the relationships between cellular responses, quantitative humoral markers, and routine laboratory parameters.

The higher cellular response observed in anti-N-seropositive participants is consistent with previous reports of enhanced SARS-CoV-2-specific immune responses in vaccinated individuals with evidence of previous viral exposure [3,18,19]. Recent evidence indicates that hybrid immunity can enhance and broaden SARS-CoV-2-specific immune responses, although the magnitude and durability of humoral and cellular responses may differ [20]. Similar findings have been reported previously, including enhanced cellular responses in vaccinated individuals with evidence of prior SARS-CoV-2 exposure [18,19,20,21,22,23]. In a longitudinal study, Juhl et al. demonstrated that SARS-CoV-2 infection enhanced both cellular and humoral immune responses compared with vaccine-induced immunity alone. Importantly, they also observed lower cellular responses with increasing age, highlighting age as an important factor when interpreting SARS-CoV-2-specific cellular immunity [24]. In the present study, the association between anti-N serostatus and cellular response remained significant after adjustment for age and sex, supporting the robustness of the observed between-group difference. Nevertheless, anti-N serostatus should not be interpreted as definitive evidence of previous infection or as establishing hybrid immunity. Rather, our findings provide a population-based characterization of cellular immune responsiveness according to a serological marker of previous SARS-CoV-2 exposure. Given the cross-sectional design and the absence of detailed information on vaccination and exposure timing, the observed association cannot be interpreted as evidence of a causal effect of previous infection on cellular immunity.

An additional aspect of the present study was the assessment of the relationship between cellular and humoral immune responses. We observed moderate positive correlations between IFN-γ responses and anti-S antibody levels in both anti-N-seronegative and anti-N-seropositive participants. These findings indicate that humoral and cellular responses are partially related, while the moderate magnitude of the correlations also suggests that anti-S antibody levels do not fully capture SARS-CoV-2-specific cellular responsiveness. This interpretation is consistent with recent longitudinal evidence showing that humoral and cellular SARS-CoV-2-specific immunity may exhibit partially distinct dynamics following vaccination and breakthrough infection. Belik et al. reported that antibody responses were more strongly influenced by vaccination and Omicron breakthrough infection, whereas T-cell responses were relatively well maintained over time [25]. Thus, quantitative anti-S serology may provide information related to, but not interchangeable with, a functional measure of cellular immune response. Importantly, the association between anti-S antibody levels and cellular response remained significant after adjustment for age, sex, and anti-N serostatus, and there was no evidence that its strength differed between the two anti-N groups.

In contrast, no significant associations were observed between cellular response and anti-N antibody levels. This finding is biologically plausible, as anti-N antibodies primarily provide evidence of previous viral exposure rather than a direct measure of the magnitude or quality of the cellular immune response. Furthermore, the restricted variability of anti-N status within the predefined groups limits the usefulness of anti-N antibody levels as a continuous correlate of cellular immune function.

We did not observe robust associations between cellular immune response and routine laboratory parameters after correction for multiple testing. This is in line with previous studies showing limited or absent associations between SARS-CoV-2-specific cellular immune responses and conventional laboratory markers, including CRP, IL-6, ferritin, D-dimer, and LDH [26,27]. Although several weak associations were observed before correction, none remained statistically significant after adjustment for multiple comparisons. Overall, the present findings do not support the use of the routinely measured laboratory parameters included in this analysis as proxies for SARS-CoV-2-specific cellular immune responsiveness. Subtle associations cannot be excluded and may require larger study populations or more targeted immunological markers to be reliably detected.

Several limitations of this study should be acknowledged. First, the lack of variability in anti-S seropositivity (100% positivity in both groups) precluded binary comparisons and restricted the analysis to quantitative differences. Second, the cross-sectional design limits causal inference and prevents assessment of the temporal dynamics of immune responses. Third, cellular immune response measurements were available for only a subset of participants (n = 246), including only 46 anti-N-seronegative participants, which limited statistical power and may have introduced selection bias. In a comparison of participants with and without available OC measurements, the proportion of anti-N-seropositive participants was higher among those with OC measurements (81.3% vs. 70.5%), and the groups also differed in examination. Because the criteria for referral for OC testing were not recorded in the available database, selection into the cellular-response subgroup could not be fully assessed. In addition, the use of anti-N serostatus as a surrogate marker of previous SARS-CoV-2 exposure may have resulted in misclassification, particularly because anti-N antibodies may wane following asymptomatic or mild infection. An important limitation was the incomplete availability of vaccination- and exposure-related information. Although vaccine type and other available covariates could be considered in adjusted analyses, information on the exact number of vaccine doses and the time since the last vaccination or previous SARS-CoV-2 exposure was unavailable. These factors may influence the magnitude and persistence of SARS-CoV-2-specific immune responses and may therefore have contributed to the observed between-group differences and correlations. Finally, cellular immunity was assessed using an antigen-stimulated IFN-γ release assay without additional cytokine profiling or multiparametric flow-cytometric immunophenotyping, limiting the characterization of specific T-cell phenotypes and memory subsets.

Taken together, our findings indicate that SARS-CoV-2-specific cellular responsiveness is associated with anti-S antibody levels but is not adequately represented by routine hematological parameters. The higher IFN-γ response observed among anti-N-seropositive participants is consistent with previous observations of enhanced cellular responses following combined SARS-CoV-2 vaccination and infection. The observed associations between cellular and humoral responses provide additional information on the relationship between these components of adaptive immunity in a vaccinated population-based cohort.

## 5. Conclusions

Anti-N seropositivity was associated with a significantly higher SARS-CoV-2 antigen-stimulated IFN-γ response among vaccinated individuals, consistent with previously described enhanced cellular responses following SARS-CoV-2 exposure in vaccinated populations. Cellular and humoral immune responses were positively correlated, although the moderate strength of these associations indicates that anti-S antibody levels do not fully reflect cellular immune responsiveness. No robust associations were identified between cellular response and routine laboratory parameters after correction for multiple comparisons. After adjustment for age and sex, the association between anti-N serostatus and cellular response remained significant, supporting the robustness of the observed between-group difference. Given the cross-sectional design and the lack of detailed information on vaccination and exposure timing, the findings should be interpreted as associations rather than causal relationships. Further studies incorporating detailed vaccination and exposure histories and more comprehensive immunological assessments are warranted to better characterize determinants of SARS-CoV-2-specific cellular immunity.

## Figures and Tables

**Table 1:** Demographic, vaccination, serological, and cellular characteristics of the study population Abbreviations: IQR, interquartile range; SD, standard deviation; OC, SARS-CoV-2 antigen-stimulated IFN-γ response.

Characteristic	Overall (N = 629)	Anti-N Seronegative (N = 159)	Anti-N Seropositive (N = 470)
Age, years, mean (SD); n	51.9 (14.3); 623	56.6 (14.7); 159	50.3 (13.8); 464
Female sex, n/N (%)	332/629 (52.8)	80/159 (50.3)	252/470 (53.6)
Male sex, n/N (%)	297/629 (47.2)	79/159 (49.7)	218/470 (46.4)
Documented vaccination, n/N (%)	414/533 (77.7)	143/144 (99.3)	271/389 (69.7)
Vaccine manufacturer available, n/N (%)	401/629 (63.8)	141/159 (88.7)	260/470 (55.3)
Pfizer, n/N (%)	230/629 (36.6)	89/159 (56.0)	141/470 (30.0)
AstraZeneca, n/N (%)	84/629 (13.4)	23/159 (14.5)	61/470 (13.0)
Moderna, n/N (%)	67/629 (10.7)	25/159 (15.7)	42/470 (8.9)
Johnson & Johnson, n/N (%)	20/629 (3.2)	4/159 (2.5)	16/470 (3.4)
Vaccine dose count available, n/N (%)	205/629 (32.6)	60/159 (37.7)	145/470 (30.9)
Anti-S antibody level, median [IQR]; n	1340 [447–4250]; 629	1240 [404.5–3240]; 159	1460 [503.8–4827.5]; 470
Anti-N antibody level, median [IQR]; n	35.48 [0.91–137.50]; 625	0.08 [0.07–0.09]; 157	73.38 [22.80–160.27]; 468
Cellular response (OC) available, n/N (%)	246/629 (39.1)	46/159 (28.9)	200/470 (42.6)
Cellular response (OC), median [IQR]; n	1560.3 [548.7–3282.7]; 246	771.1 [176.2–1612.1]; 46	1752.3 [658.4–3791.2]; 200

## Data Availability

The data supporting the findings of this study can be obtained from the corresponding author upon reasonable request.
